# Synthesis of Novel *bis*-1,5-Disubstituted-1*H*-Tetrazoles by an Efficient Catalyst-Free Ugi-Azide Repetitive Process

**DOI:** 10.3390/molecules20011519

**Published:** 2015-01-16

**Authors:** Luís E. Cárdenas-Galindo, Alejandro Islas-Jácome, Karla M. Colmenero-Martínez, Antonio Martínez-Richa, Rocío Gámez-Montaño

**Affiliations:** 1 Departamento Química Área Ambiental, Universidad Tecnológica de Salamanca, Av. Universidad Tecnológica No. 200, Col. Ciudad Bajío, Salamanca C.P. 36750, Gto., Mexico; E-Mail: lcardenas@utsalamanca.edu.mx; 2 Departamento de Química, División de Ciencias Naturales y Exactas, Universidad de Guanajuato, Noria Alta S/N, Col. Noria Alta, Guanajuato C.P. 36050, Gto., Mexico; E-Mails: blackheim66@gmail.com (A.I.-J.); karlasol08@hotmail.com (K.M.C.-M.); richa@ugto.mx (A.M.-R.)

**Keywords:** *bis*-1,5-disubstituted-1*H*-tetrazoles, Ugi-azide repetitive reaction, MW-assisted synthesis, catalyst-free, chelating agents

## Abstract

A series of five novel *bis*-1,5-disubstituted-1*H*-tetrazoles (*bis*-1,5-DS-1*H*-T) were quickly prepared by a catalyst-free Ugi-azide repetitive process from easily accessible starting materials in excellent yields, either at room temperature (88%–95%) or using mild MW-heating conditions (80%–91%). These molecules may have a wide range of applications, such as chelating agents, organocatalysts and luminescent materials, and mainly as bioactive compounds.

## 1. Introduction

**Figure 1:**
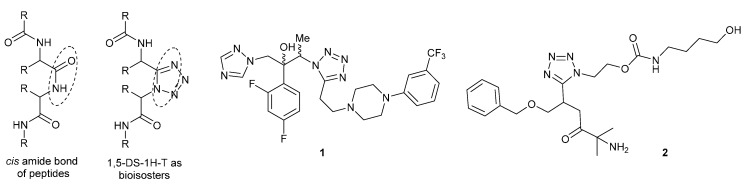
Some representative 1,5-disubstituted-1*H*-tetrazoles (1,5-DS-1*H*-T) having biological activity.

1,5-Disubstituted-1*H*-tetrazoles (1,5-DS-1*H*-T) are a special kind of non-naturally occurring heterocycles having a wide range of applications, mainly in medicinal chemistry, since they have proven to be suitable bioisosteres of the *cis*-amide bonds of peptides, because of the capability to adopt their conformations [1]. For example, the 1,5-DS-1*H*-T **1** showed antifungal activity against *Candida albicans*, *Cryptococcus neoformans* and *Aspergillus niger* comparable to the most potent commercially available antimycotics, such as fluconazole and itraconazole [2]. In the same context, the in-clinical phase drug, BMS-317180 (**2**), is a potent orally agonist of the human growth hormone, secretagogue [3] (Figure 1). It is noteworthy that the most used methods toward the synthesis of the 1,5-DS-1*H*-T are both click [3 + 2] dipolar cycloadditions of organic azides with cyanides [4,5,6,7] and the Ugi-azide reaction between amines, aldehydes, isocyanides and hydrazoic acid [8].

**Figure 2:**
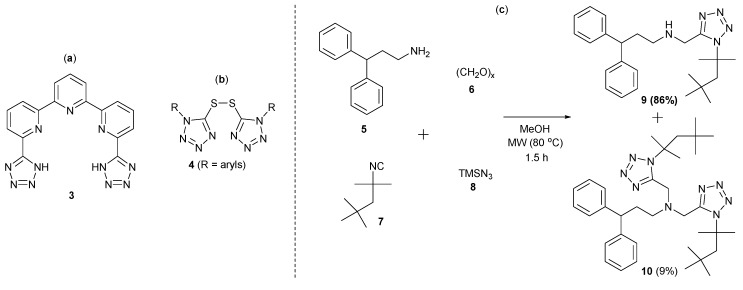
(**a**) Lanthanide cations ligand **3**; (**b**) bioactive *bis*-1,5-DS-1*H*-T **4**; (**c**) synthesis of the *bis*-1,5-DS-1*H*-T **10** as a by-product of a Ugi-azide reaction.

The synthesis, biological activity and other applications of molecules having two or more tetrazole rings in their structures have been reported little in the literature. Particularly, the *bis*-1,5-DS-1*H*-T attracted our attention, since they have shown interesting properties. Mazzanti and co-workers reported the use of the terpyridine linear-based *bis*-tetrazole **3** as a polydentate ligand of lanthanide cations, such as Eu^III^, Tb^III^ and Nd^III^, allowing the synthesis of various potent luminescent materials [9]. The *bis*-sulfide-tetrazoles **4**, which were biologically tested by Waisser and co-workers, showed a strong antiulcer, as well as antimycobacterial activity [10]. With the intention of preparing aminomethyltetrazole **9** (85%) as an inhibitor of the γ-aminobutyric acid transporters, mGAT1-mGAT4, Wanner and co-workers synthesized *bis*-1,5-DS-1*H*-T **10** (9%) as a by-product of an Ugi-azide reaction from amine **5**, paraformaldehyde (**6**), isocyanide **7** and azidotrimethylsilane (**8**) [11] (Figure 2).

Thus, based on our ongoing efforts in the development of short and versatile methodologies toward the synthesis of tetrazole-containing hybrid compounds having frameworks of interest in medicinal chemistry, such as terazol-chromones [12], tetrazol-tetrahydro-β-1*H*-carbolines [13] and tetrazol-azepinoindolones [14], we herein report the synthesis of a series of five novel *bis*-1,5-DS-1*H*-T **15a**–**e** using a catalyst-free Ugi-azide repetitive process.

## 2. Results and Discussion

**Scheme 1:**
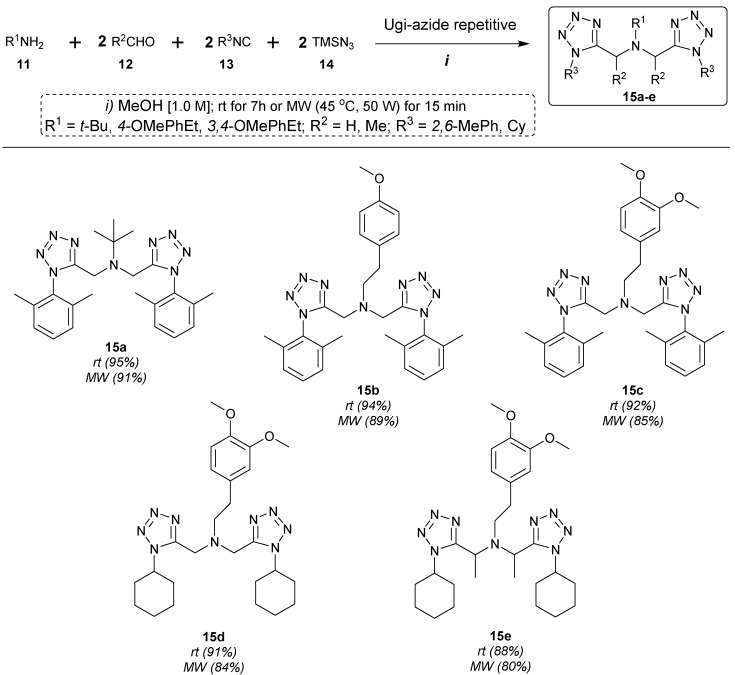
Synthesis of *bis*-1,5-DS-1*H*-T **15a**–**e**.

After optimizing the reaction conditions, the series of novel *bis*-1,5-DS-1*H*-T **15a**–**e** were prepared by a sequential combination of one equivalent of amines **11**, with two equivalents of aldehydes **12**, isocyanides **13** and the azidotrimethylsilane **14** in MeOH (1.0 M) at room temperature under catalyst-free conditions in seven hours of reaction (Scheme 1). It is noteworthy that excellent yields were obtained (88%–95%). Compound **4a** was synthesized with the best yield (95%) when *tert-*butylamine (**11a**), paraformaldehyde (**12a**), 2,6-dimethylphenyl isocyanide (**13a**) and azidotrimethylsilane (**14**) were reacted together. This highest yield was observed possibly due to the high nucleophilicity of the *tert*-butylamine compared with the others amines used to complete the series. Then, with the intention of studying the effects coming from the amine moiety, the *bis*-1,5-DS-1*H*-T **15b**–**c** were prepared also in excellent yields (94% and 92%, respectively) by using amines with different stereoelectronic natures. Then, making a comparison between the yields of products **15a**–**c** (2,6-dimethylphenylisocyanide derivatives) and **15d**–**e** (cyclohexyl isocyanide derivatives), it can be seen that the last ones were obtained in slightly lower yields, possibly due to the difference in the electronic nature coming just from the isocyanide substituent, which can affect the reaction course. One last interesting replacement was done when acetaldehyde was used instead of paraformaldehyde to prepare Compound **15e**. As can be seen, the observed yield for this latter (88%) is not much different with respect to the others despite the volatility of the acetaldehyde. Finally, with the intention of decreasing the reaction times, we used mild MW heating conditions, and slightly lower yields were observed with respect to those obtained at room temperature, but in only 15 minutes of reaction. It is noteworthy that the relationship of yields at room temperature and MW was kept. Compound **15a** was obtained with the highest yield (91%) and **15e** with the lowest yield (80%) (Scheme 1).

## 3. Experimental Section

^1^H- and ^13^C-NMR spectra were acquired on Bruker Advance II spectrometers (200 or 300 MHz). The solvent was CDCl_3_. Chemical shifts are reported in parts per million (δ/ppm). The internal reference for ^1^H-NMR spectra is with respect to tetramethylsilane (TMS) at 0.0 ppm. The internal reference for ^13^C-NMR spectra is with respect to CDCl_3_ at 77.0 ppm. Coupling constants are reported in Hertz (*J*/Hz). Multiplicities of the signals are reported using the standard abbreviations: singlet (s), doublet (d), triplet (t), quartet (q) and multiplet (m). IR spectra were recorded on a Perkin Elmer 100 FTIR spectrometer using neat compounds, and the wavelengths are reported in reciprocal centimeters (ν/cm^−1^). HRMS spectra were acquired on a JEOL JEM-AX505HA spectrometer, and the samples were ionized by FAB+ and recorded via the TOF method. Microwave-assisted reactions were performed in closed vessel mode using a monomodal CEM Discover unit. The reaction progress was monitored by TLC, and the spots were visualized under UV light at 254 or 365 nm. Flash column chromatography was performed using silica gel (230–400 mesh) and a mixture of hexanes with AcOEt (4:1 v/v) as the mobile phase. Melting points were determined on a Fisher-Johns apparatus and were uncorrected. All commercially available starting materials were used without further purification. The solvents were distilled and dried according to standard procedures. The NMR spectra of Compounds **15a**–**e** are shown in the Supplementary Material.

*General Procedures for the Synthesis of bis-1,5-Disubstituted-1H-tetrazoless* **15a**–**e**

General Procedure 1 (GP-1) (room temperature conditions): In a round-bottomed flask equipped with a magnetic stirrer bar, to a 1.0 M solution of amine **11** (1.0 equiv) in anhydrous MeOH under nitrogen atmosphere, aldehyde **12** (2.0 equiv), isocyanide **13** (2.0 equiv) and azidotrimethylsilane (**14**) (2.0 equiv) were sequentially added. The resulting mixture was stirred at room temperature for 7 h. Then, the solvent was removed until dryness. The crude extract was diluted with CH_2_Cl_2_ (20 mL) and washed with an excess of brine. The aqueous layer was extracted with CH_2_Cl_2_ (2 × 10 mL). The combined organic layer was dried over anhydrous Na_2_SO_4_, evaporated until dryness and purified by silica gel column chromatography using a mixture of hexanes with ethyl acetate (4:1 v/v) as the mobile phase to afford the corresponding *bis*-1,5-disubstituted-1*H*-tetrazoles **15a**–**e**.

General Procedure 2 (GP-2) (microwave heating conditions): In a MW sealed tube equipped with a magnetic stirring bar, to a 1.0 M solution of amine **11** (1.0 equiv) in anhydrous MeOH, aldehyde **12** (2.0 equiv), isocyanide **13** (2.0 equiv) and azidotrimethylsilane (**14**) (2.0 equiv) were sequentially added. The resulting mixture was MW-heated 3 times for 5 minutes at 45 °C (50 W). Then, the solvent was removed until dryness. The crude extract was diluted with CH_2_Cl_2_ (20 mL) and washed with an excess of brine. The aqueous layer was extracted with CH_2_Cl_2_ (2 × 10 mL). The combined organic layer was dried over anhydrous Na_2_SO_4_, evaporated until dryness and purified by silica gel column chromatography using a mixture of hexanes with ethyl acetate (4:1 v/v) as the mobile phase to afford the corresponding *bis*-1,5-disubstituted-1*H*-tetrazoles **15a**–**e**.

*N,N-bis((1-(2,6-Dimethylphenyl)-1H-tetrazol-5-yl)methyl)-2-methylpropan-2-amine* (**15a**). *t*-Butylamine (150 mg, 2.05 mmol), paraformaldehyde (123 mg, 4.10 mmol), 2,6-dimethylphenyl isocyanide (538 mg, 4.10 mmol) and azidotrimethylsilane (473 mg, 4.10 mmol) were reacted together in anhydrous MeOH (2.1 mL) to afford Compound **15a** (866 mg, 95%, GP-1; 829 mg, 91%, GP-2) as a white powder; mp = 132–134 °C; R*_f_* = 0.48 (Hex/AcOEt 4:1 v/v); FTIR (Attenuated Total Reflection, ATR) ν_max_/cm^−1^ 776, 784, 845, 929, 1035, 1112, 1086, 1196, 1369, 1483, 1719, 2963; ^1^H-NMR (300 MHz; CDCl_3_; 25 °C; TMS): δ 0.87 (s, 9H), 1.98 (s, 12H), 4.06 (s, 4H), 7.24 (d, 4H, *J* = 7.4 Hz), 7.39 (dd, 2H, *J* = 8.2, 7.0 Hz); ^13^C-NMR (75 MHz, CDCl_3_; 25 °C; TMS): δ 17.7, 27.6, 41.1, 56.0, 129.1, 131.1, 131.9, 136.0, 154.8; HRMS [M−H]^+^ *m/z* calcd. for C_24_H_32_N_9_^+^ 446.2781, found 446.2775.

*N,N-bis((1-(2,6-Dimethylphenyl)-1H-tetrazol-5-yl)methyl)-2-(4-methoxyphenyl)ethan-1-amine* (**15b**). 4-Methoxyphenethylamine (300 mg, 1.98 mmol), paraformaldehyde (119 mg, 3.97 mmol), 2,6-dimethylphenyl isocyanide (521 mg, 3.97 mmol) and azidotrimethylsilane (457 mg, 3.97 mmol) were reacted together in MeOH (2.0 mL) to afford Compound **15b** (973 mg, 94%, GP-1; 921 mg, 89%, GP-2) as a white powder; mp = 154–156 °C; R*_f_* = 0.23 (Hex/AcOEt 4:1 v/v); FTIR (ATR) ν_max_/cm^−1^ 779, 1031, 1104, 1123, 1243, 1452, 1481, 1509, 1608, 2150, 2834, 2928; ^1^H-NMR (200 MHz; CDCl_3_; 25 °C; TMS): δ 1.87 (s, 12H), 2.32 (dd, 2H, *J* = 9.2, 6.6 Hz), 2.90 (dd, 2H, *J* = 9.3, 6.6 Hz), 3.75 (s, 3H), 3.92 (s, 4H), 6.74 (d, 2H, *J* = 8.6 Hz), 6.86 (d, 2H, *J* = 8.6 Hz), 7.21 (d, 4H, *J* = 7.6 Hz), 7.38 (dd, 2H, *J* = 8.3, 6.9 Hz); ^13^C-NMR (50 MHz, CDCl_3_; 25 °C; TMS): δ 17.5, 32.9, 45.3, 55.3, 56.1, 114.0, 129.1, 129.4, 130.7, 131.2, 131.7, 135.7, 152.9, 158.2; HRMS [M−H]^+^ *m/z* calcd. for C_29_H_34_ON_9_^+^ 524.2886, found 524.2885.

*2-(3,4-Dimethoxyphenyl)-N,N-bis((1-(2,6-dimethylphenyl)-1H-tetrazol-5-yl)methyl)ethan-1-amine* (**15c**). 3,4-Dimethoxyphenethylamine (500 mg, 2.76 mmol), paraformaldehyde (166 mg, 5.52 mmol), 2,6-dimethylphenyl isocyanide (724 mg, 5.52 mmol) and azidotrimethylsilane (636 mg, 5.52 mmol) were reacted together in MeOH (2.8 mL) to afford Compound **15c** (1405 mg, 92%, GP-1; 1298 mg, 85%, GP-2) as a yellow powder; mp = 48–50 °C; R*_f_* = 0.49 (Hex-AcOEt 4:1 v/v); FTIR (ATR) ν_max_/cm^−1^ 764, 1027, 1103, 1140, 1235, 1235, 1260, 1417, 1464, 1514, 1590, 2835, 2856, 2929; ^1^H-NMR (300 MHz; CDCl_3_; 25 °C; TMS): δ 1.86 (s, 12H), 2.36 (m, 2H), 2.94 (m, 2H), 3.82 (s, 6H), 3.92 (s, 4H), 6.46–6.52 (m, 2H), 6.70 (d, 1H, *J* = 8.1 Hz), 7.21 (d, 4H, *J* = 7.6 Hz), 7.38 (m, 2H); ^13^C-NMR (75 MHz, CDCl_3_; 25 °C; TMS): δ 17.4, 33.3, 45.4, 55.8, 56.0, 111.5, 111.9, 120.4, 128.9, 129.0, 131.1, 131.4, 131.6, 135.7, 147.7, 149.1, 152.9; HRMS [M−H]^+^ *m/z* calcd. for C_30_H_35_O_2_N_9_^+^ 554.2992, found 554.2981.

*N,N-bis((1-Cyclohexyl-1H-tetrazol-5-yl)methyl)-2-(3,4-dimethoxyphenyl)ethan-1-amine* (**15d**). 3,4-Dimethoxyphenethylamine (300 mg, 1.66 mmol), paraformaldehyde (99 mg, 3.31 mmol), cyclohexyl isocyanide (361 mg, 3.97 mmol) and azidotrimethylsilane (381 mg, 3.31 mmol) were reacted together in MeOH (1.7 mL) to afford Compound **15d** (768 mg, 91%, GP-1; 708 mg, 84%, GP-2) as a white powder; mp = 126–127 °C; R*_f_* = 0.42 (Hex/AcOEt 4:1 v/v); FTIR (ATR) ν_max_/cm^−1^ 782, 1033, 1119, 1108, 1227, 1463, 1492, 1521, 1615, 2129, 2851, 2935; ^1^H-NMR (200 MHz; CDCl_3_; 25 °C; TMS): δ 1.19–1.34 (m, 7H), 1.82–1.90 (m, 13H), 2.73 (t, 2H, *J* = 7.4 Hz), 2.93 (t, 2H, *J* = 7.4 Hz), 3.74 (s, 3H), 3.76 (s, 3H), 3.98 (s, 4H), 4.07–4.15 (m, 2H), 6.58 (d, 2H, *J* = 7.7 Hz), 6.75 (d, 1H, *J* = 7.8 Hz); ^13^C-NMR (50 MHz, CDCl_3_; 25 °C; TMS): δ 24.9, 25.2, 32.9, 46.0, 56.1, 57.9, 111.4, 111.9, 120.5, 131.4, 147.8, 149.2, 150.9; HRMS [M−H]^+^ *m/z* calcd. for C_26_H_39_N_9_O_2_ 510.3299, found 510.3298.

*1-(1-Cyclohexyl-1H-tetrazol-5-yl)-N-(1-(1-cyclohexyl-1H-tetrazol-5-yl)ethyl)-N-(3,4-dimethoxyphenethyl)ethan-1-amine* (**15e**). 3,4-Dimethoxyphenethylamine (300 mg, 1.66 mmol), acetaldehyde (146 mg, 3.31 mmol), cyclohexyl isocyanide (361 mg, 3.31 mmol) and azidotrimethylsilane (381 mg, 3.31 mmol) were reacted together in MeOH (1.7 mL) to afford Compound **15e** (783 mg, 88%, GP-1; 711, 80%, GP-2) as a white powder; mp = 115–117 °C; R*_f_* = 0.46 (Hex/AcOEt 4:1 v/v); FTIR (ATR) ν_max_/cm^−1^ 749, 759, 808, 1027, 1048, 1233, 1263, 1456, 1,586, 1512, 1672, 2858, 2935, 3339; ^1^H-NMR (300 MHz; CDCl_3_; 25 °C; TMS): δ 1.28 (d, 6H, *J* = 6.9 Hz), 1.44–1.60 (m, 4H), 1.78–2.05 (m, 12H), 2.12–2.19 (m, 4H), 2.74 (m, 2H), 3.33 (m, 2H), 3.87 (s, 3H), 3.89 (s, 3H), 4.23 (q, 2H, *J* = 6.9 Hz), 4.39–4.49 (m, 2H), 6.66 (d, 1H, *J* = 1.9 Hz), 6.72 (dd, 1H, *J* = 8.1, 1.9 Hz), 6.81 (d, 1H, *J* = 8.1 Hz); ^13^C-NMR (75 MHz, CDCl_3_; 25 °C; TMS): δ 17.2, 25.0, 25.5, 25.5, 32.7, 34.0, 36.9, 47.9, 48.4, 56.2, 56.2, 57.8, 111.7, 112.1, 120.7, 131.6, 148.0, 149.3, 155.2; HRMS [M−H]^+^ *m/z* calcd. for C_28_H_44_O_2_N_9_^+^ 538.3618, found 538.3629.

## 4. Conclusions

A series of five novel *bis*-1,5-disubstituted-1*H*-tetrazoles (*bis*-1,5-DS-1*H*-T) were quickly prepared by a catalyst-free Ugi-azide repetitive process from easily accessible starting materials in excellent yields, either at room temperature (88%–95%) or using mild MW-heating conditions (80%–91%). The yields are slightly dependent on stereo-electronic factors. The methodology herein described could be considered as a pioneering work in the one pot synthesis of *bis*-1,5-DS-1*H*-T, which, in fact, could present a wide range of applications, such as luminescent materials, anionic chelating agents or bioactive compounds.

## Supplementary Materials

Supplementary materials can be accessed at: http://www.mdpi.com/1420-3049/20/01/1519/s1.
