# Supplementary material for: Synthesis of Novel bis-1,5-Disubstituted-1H-Tetrazoles by an Efficient Catalyst-Free Ugi-Azide Repetitive Process
Source: Molecules. 2015 Jan 16;20(1):1519–26. doi: 10.3390/molecules20011519 (PMC6272218; doi:10.3390/molecules20011519)
Supplement: Supplementary file 1 [file molecules-20-01519-s001.pdf]

Supplementary Materials

Contents

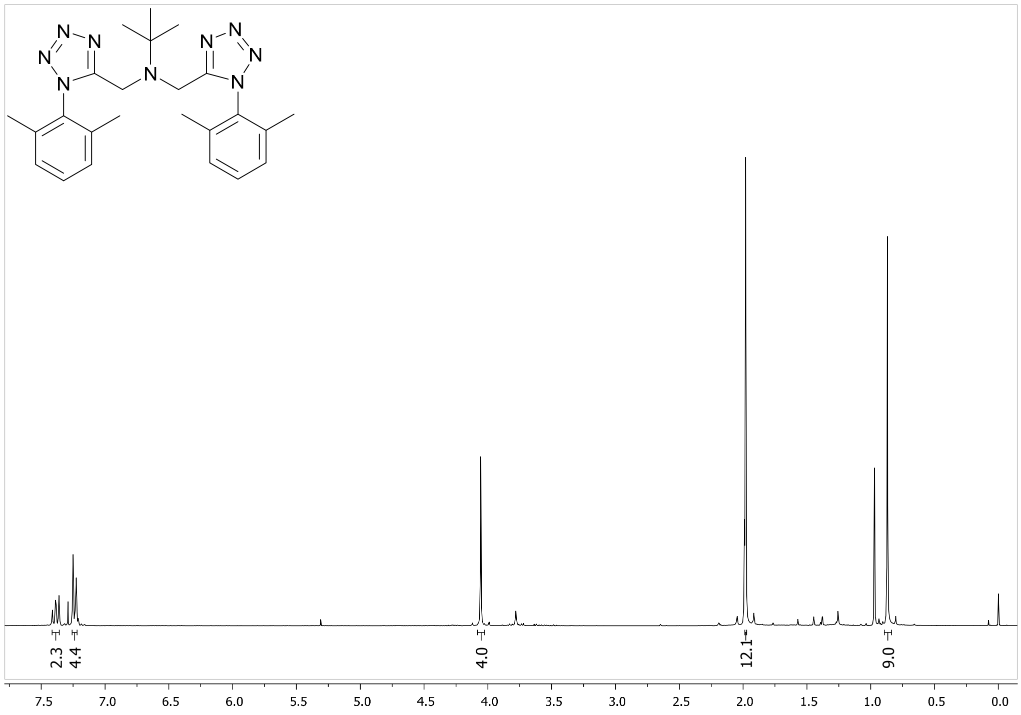

Figure S1. ^{1}H-NMR spectra of Compound **15a**.

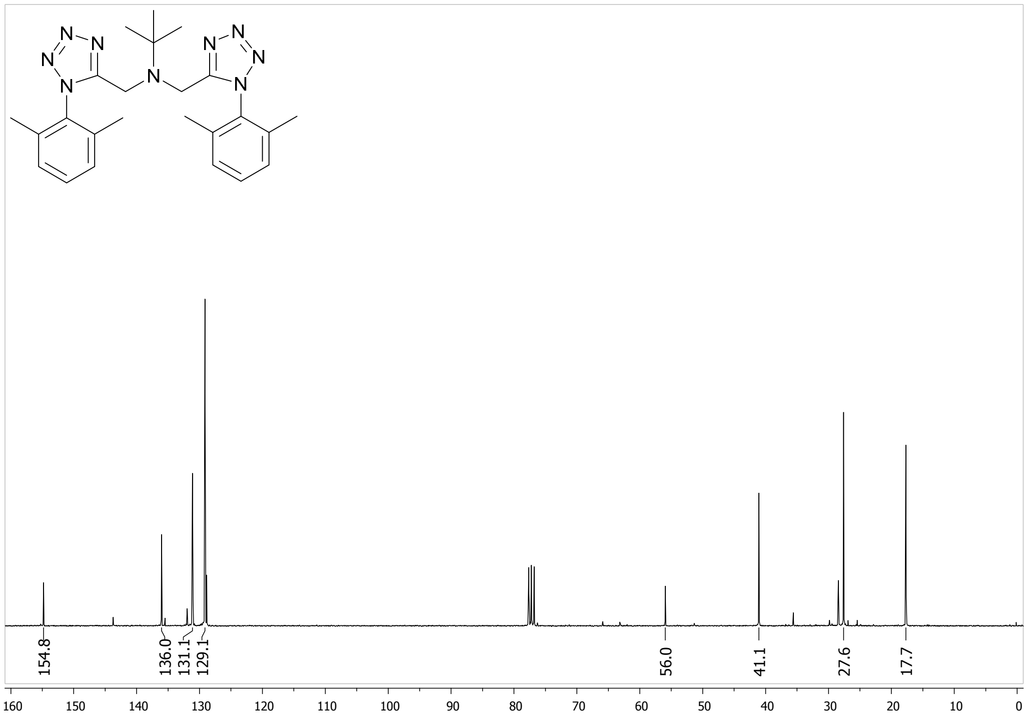

Figure S2. ^{13}C-NMR spectra of Compound **15a**.

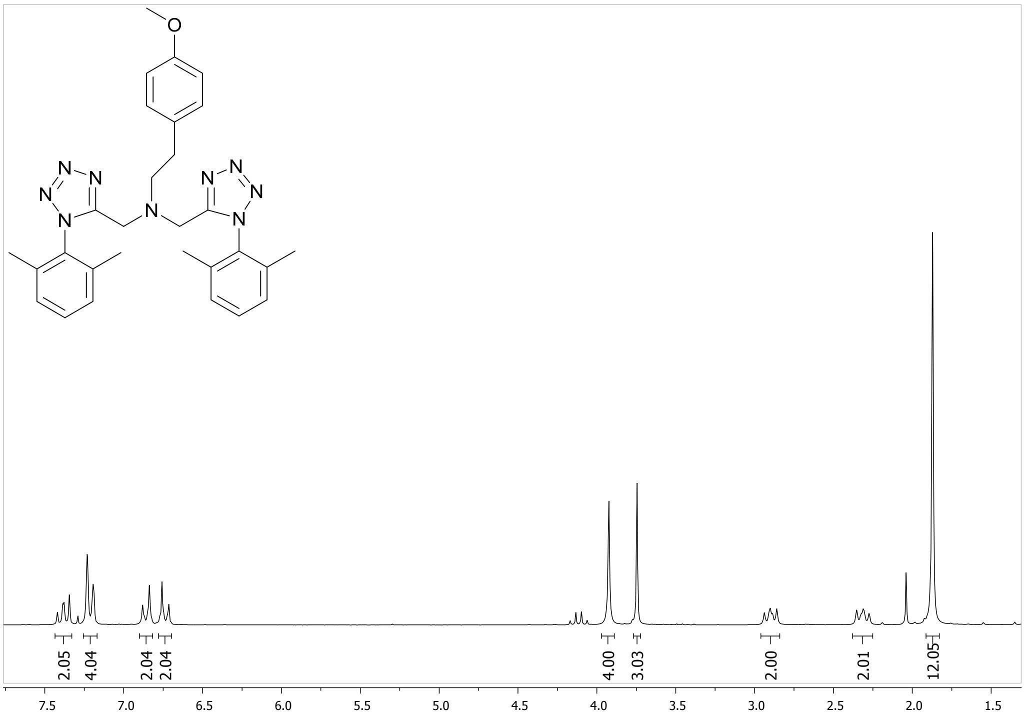

Figure S3. ^{1}H-NMR spectra of Compound **15b**.

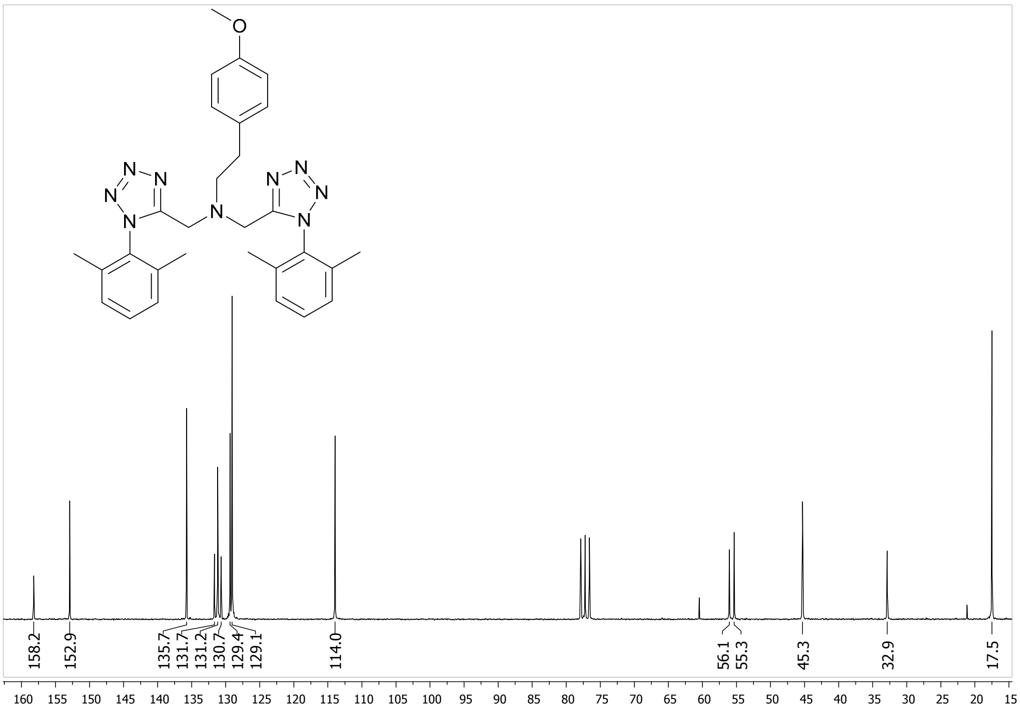

Figure S4. ^{13}C-NMR spectra of Compound **15b**.

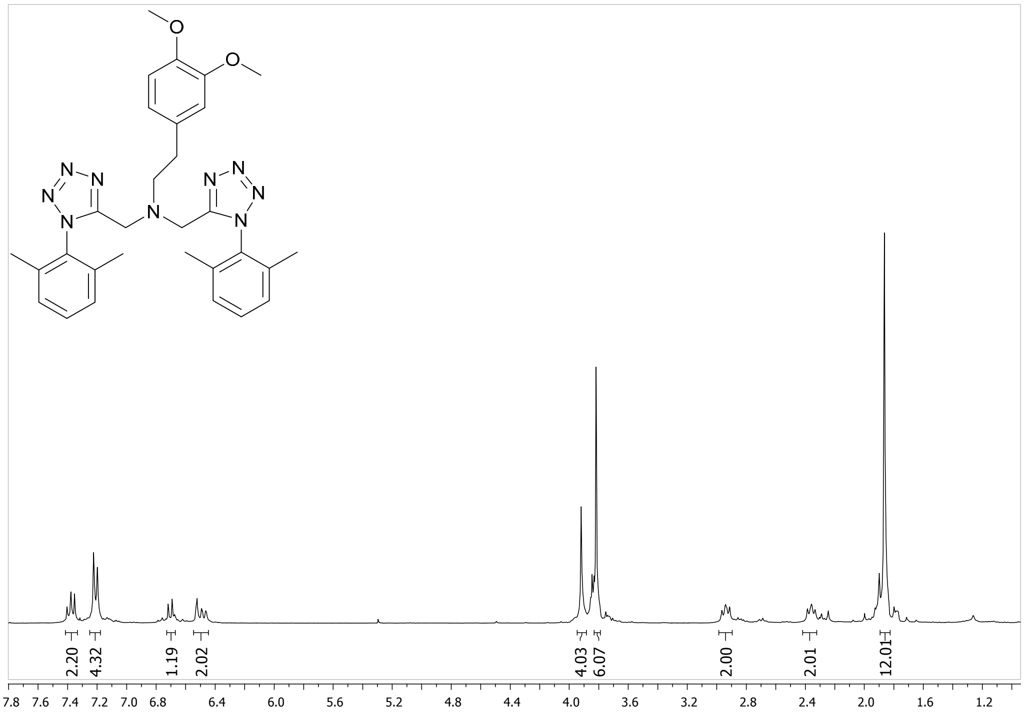

Figure S5. ^{1}H-NMR spectra of Compound **15c**.

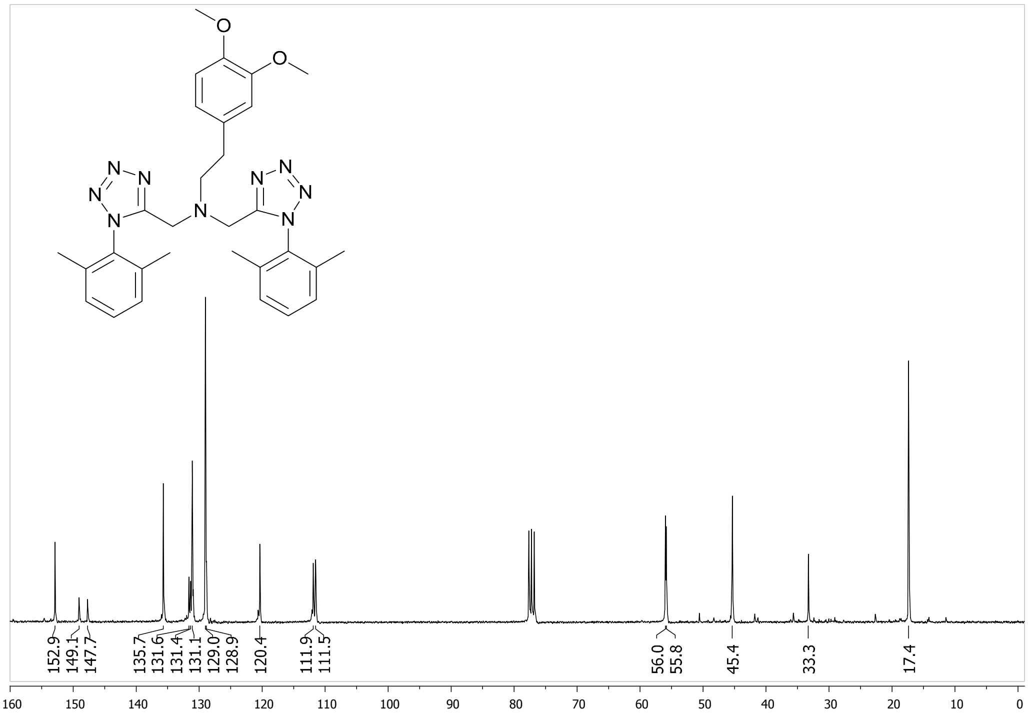

Figure S6. ^{13}C-NMR spectra of Compound **15c**.

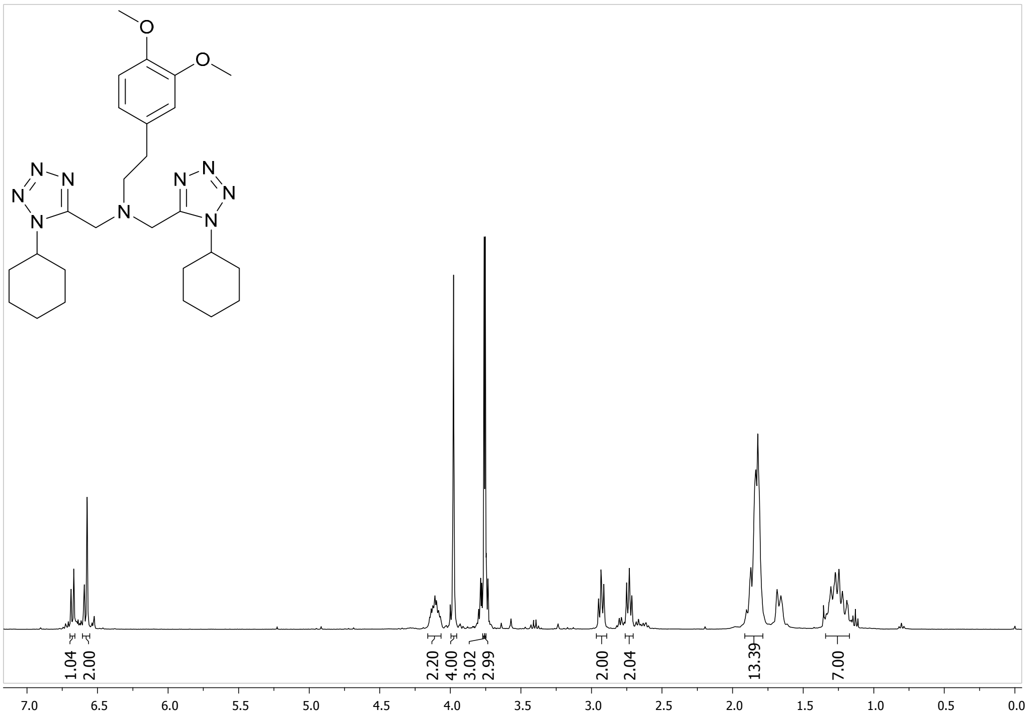

Figure S7. ^{1}H-NMR spectra of Compound **15d**.

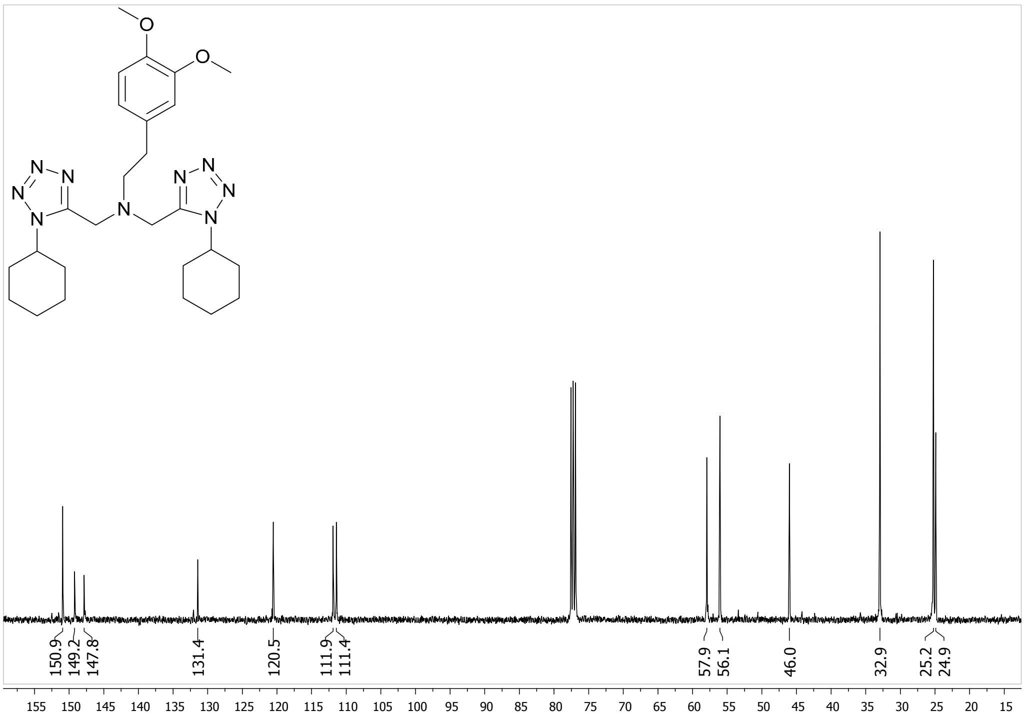

Figure S8. ^{13}C-NMR spectra of Compound **15d**.

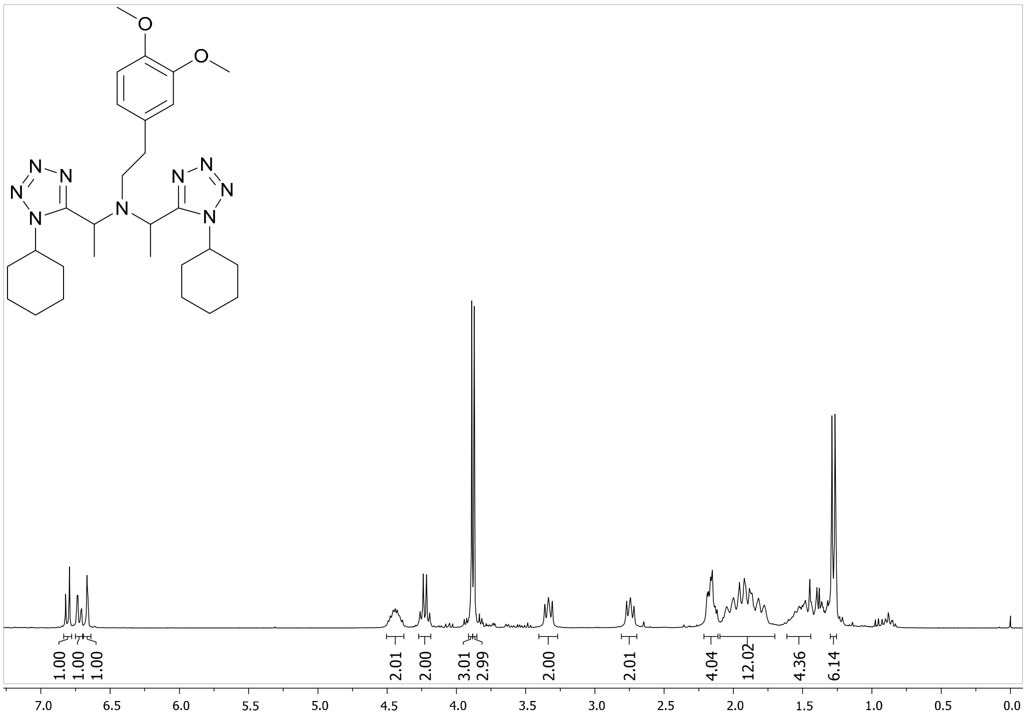

Figure S9. ^{1}H-NMR spectra of Compound **15e**.

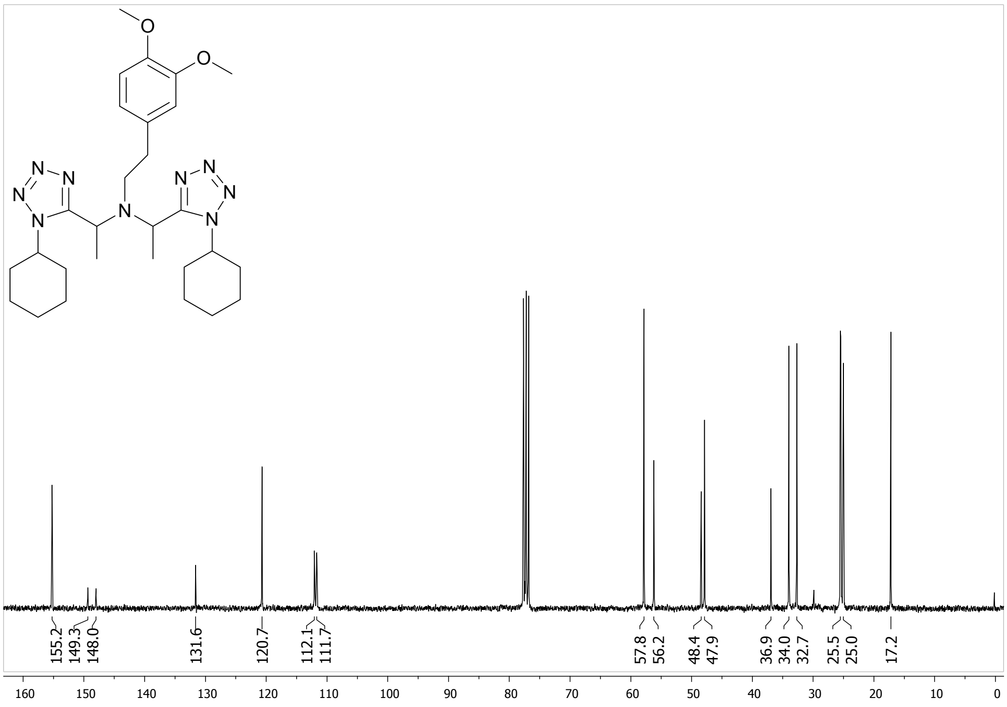

Figure S10. ^{13}C-NMR spectra of Compound **15e**.
